# Neural correlates of emotional valence for faces and words

**DOI:** 10.3389/fpsyg.2023.1055054

**Published:** 2023-02-23

**Authors:** Daniela Ballotta, Riccardo Maramotti, Eleonora Borelli, Fausta Lui, Giuseppe Pagnoni

**Affiliations:** ^1^Department of Biomedical, Metabolic and Neural Sciences, University of Modena and Reggio Emilia, Modena, Italy; ^2^Department of Medical and Surgical, Maternal-Infantile and Adult Sciences, University of Modena and Reggio Emilia, Modena, Italy

**Keywords:** fMRI, emotion, facial expression, faces, words, valence, dynamic causal modeling

## Abstract

Stimuli with negative emotional valence are especially apt to influence perception and action because of their crucial role in survival, a property that may not be precisely mirrored by positive emotional stimuli of equal intensity. The aim of this study was to identify the neural circuits differentially coding for positive and negative valence in the implicit processing of facial expressions and words, which are among the main ways human beings use to express emotions. Thirty-six healthy subjects took part in an event-related fMRI experiment. We used an implicit emotional processing task with the visual presentation of negative, positive, and neutral faces and words, as primary stimuli. Dynamic Causal Modeling (DCM) of the fMRI data was used to test effective brain connectivity within two different anatomo-functional models, for the processing of words and faces, respectively. In our models, the only areas showing a significant differential response to negative and positive valence across both face and word stimuli were early visual cortices, with faces eliciting stronger activations. For faces, DCM revealed that this effect was mediated by a facilitation of activity in the amygdala by positive faces and in the fusiform face area by negative faces; for words, the effect was mainly imputable to a facilitation of activity in the primary visual cortex by positive words. These findings support a role of early sensory cortices in discriminating the emotional valence of both faces and words, where the effect may be mediated chiefly by the subcortical/limbic visual route for faces, and rely more on the direct thalamic pathway to primary visual cortex for words.

## 1. Introduction

The ability to recognize other people’s emotions is crucial for effective social interactions, where emotional content is predominantly conveyed by facial expressions and words (when words are spoken, rather than read, an essential additional factor is the tonal features of the utterance). For basic emotions, the generation and processing of facial expressions of emotions has been shown to be universal (Nelson et al., 1979; Adolphs, 2002; Cowen et al., 2021; but see different views in, e.g., Hoemann et al., 2019; Berent et al., 2020), with a processing advantage for emotional faces compared to neutral ones (Johansson et al., 2004; Gross and Schwarzer, 2010). More specifically, a prioritization of negative stimuli has been inferred by faster processing times (Fox et al., 2000) and a stronger effect of attentional capture (Alpers and Gerdes, 2007; Bach et al., 2014). An enhancement of perceptual encoding has been observed not only for those stimuli that are recognizable at a very young age (Nelson et al., 1979), but also for stimuli whose affective valence is learned later in life, such as emotional words (Kanske and Kotz, 2007; Kousta et al., 2009; Yap and Seow, 2014; Goh et al., 2016).

Over the past two decades, the neuronal substrates of emotional processing have represented an intensely researched area in the field of affective neurosciences. Even though functional magnetic resonance imaging (fMRI) studies have demonstrated that the processing of facial and linguistic expressions is supported by two different distributed neural networks (Haxby et al., 2002; Price, 2012), the existence of a common brain substrate underlying general emotional processing has also been hypothesized. In fact, an enhancement of blood oxygen level-dependent (BOLD) signal in visual cortices has been reported for various types of emotional, as opposed to non-emotional, stimuli, including faces (Vuilleumier and Schwartz, 2001; Reisch et al., 2020), pictures of complex scenes (Aldhafeeri et al., 2012) and words (Herbert et al., 2009; Schlochtermeier et al., 2013; Hoffmann et al., 2015).

Nonetheless, while the emotion-related effect of faces in visual processing areas is well supported by the literature, the importance of early sensory cortices for emotion-related word processing is more controversial. An electroencephalographic (EEG) study showed that even when emotion is irrelevant for the task (e.g., in a simple face-word discrimination task), the sensory encoding of emotional content is automatically enhanced for face but not for word stimuli (Rellecke et al., 2011). Similarly, a robust overlapping activation pattern in the extrastriate visual cortex was reported in meta-analytic fMRI studies for pictorial emotional stimuli of different types, but this overlap did not extend to words (Sabatinelli et al., 2011; García-García et al., 2016). A recent fMRI study, directly comparing negative faces, pictures, and words, confirmed the relevance of extrastriate visual areas for faces and pictures, and of left-lateralized frontal and parietal semantic processing areas for lexical stimuli (Reisch et al., 2020).

On the other hand, the importance of early sensory brain cortices for affective experience has been claimed by several studies suggesting the existence of a sensory, modality-specific representation of emotional valence (Satpute et al., 2015; Miskovic and Anderson, 2018). Besides the involvement of early sensory cortices, specific emotion effects for faces were also reported in the “core network” of face processing (Haxby et al., 2000, 2002), including fusiform gyrus, superior temporal sulcus, and inferior occipital cortex (Sabatinelli et al., 2011).

Emotion processing has also been linked to the activity of subcortical brain structures. Contemporary theories ascribe a key role to the amygdala in contextually evaluating and integrating a variety of sensory informations, “tagging” them with appropriate values of emotional dimensions (valence, intensity, and approachability; for a review see Šimić et al., 2021). The amygdala has traditionally been implicated in the modulation of sensory responses to emotional stimuli, particularly fearful ones, and it is known to be anatomically connected to visual cortical regions (LeDoux, 1996; Gschwind et al., 2012). In fact, visual emotional signals appear to be processed by two parallel pathways, both involving the amygdala, albeit at different processing stages: a cortical “high road,” and a subcortical “low road.” The first route encompasses the thalamic lateral geniculate nucleus, the striate cortex, and the amygdala, and it allows fine-grained, but slow, evaluations of the stimuli. In the second, subcortical route, visual information proceeds to the superior colliculus before being relayed to the amygdala *via* the pulvinar, and it allows for a fast, but coarse, analysis of the visual input, including potential threat (LeDoux, 1996). A meta-analysis of fMRI studies has demonstrated the involvement of the amygdala for both positive and negative facial expressions (Fusar-Poli et al., 2009), confirming its relevance in the processing of affective content, regardless of valence. The amygdala is also implicated in emotional word processing. It has been shown to rapidly respond to emotional words at an early stage of reading (200 ms after stimulus onset; Naccache et al., 2005), similarly to the occipitotemporal visual word form area (Gaillard et al., 2006), which mediates orthographic processing (Cohen et al., 2000). Finally, a functional coupling between the amygdala and the extrastriate cortex was described in both the left (Herbert et al., 2009) and the right hemisphere (Tabert et al., 2001) during reading of affective words.

Recent studies have applied Dynamic Causal Modeling (DCM) (Friston et al., 2003) — a Bayesian statistical framework to assess effective connectivity in the brain — to examine directional influences within the face processing network (Fairhall and Ishai, 2007; Torrisi et al., 2013; Jamieson et al., 2021). Initially, a hierarchical structure of the “core” and “extended” face networks was proposed, where the “core” network (including mainly visual areas) was hypothesized to exert a feed-forward, bottom-up influence on the “extended” network (prefrontal and limbic regions), considered to be responsible for emotional and social aspects (Fairhall and Ishai, 2007; Da Silva et al., 2011; Torrisi et al., 2013; Sladky et al., 2015). On the other hand, a top-down, feedback effect of the amygdala on visual areas (e.g., occipital face area and fusiform gyrus) was reported by Furl et al. (2013), suggesting that the amygdala mediates an optimization of visual processing depending on the emotional valence of the stimulus. An affective modulation of bidirectional connections between frontal and subcortical structures has also been described. Willinger et al. (2019) showed a modulation of top-down connectivity between the medial prefrontal cortex and the amygdala by positive and negative faces, whereas the bottom-up connectivity between the same regions was modulated by negative and neutral faces only. An increased bidirectional connectivity between frontal areas and the amygdala was also observed during an emotional learning task involving the pairing of emotional words and pictures (Ćurcić-Blake et al., 2012); the modulation was associated with emotional (in particular, negative) stimuli. In summary, as suggested by the review of Underwood et al. (2021), DCM findings suggest a model of emotional processing characterized by dynamic and largely bidirectional modulatory relationships between cortical and subcortical regions.

The aim of the present study was to better understand the functional organization of emotion processing networks, identifying brain circuits coding for valence in the implicit processing of visual stimuli. We chose facial expressions and words as common instances of largely innate and culturally acquired stimuli, respectively (for a recent proposal arguing against this dichotomy, see, however, Feldman Barrett, 2017). The mechanisms by which emotional stimuli may trigger different behavioral reactions (e.g., related to the basic dimensions of approach and avoidance) and attentional movements are particularly relevant for social interactions. Several studies suggest that the neural pathways processing innate and acquired negative valence, as well as the associated avoidance behaviors, may be different (Kim and Jung, 2006; Isosaka et al., 2015; Silva et al., 2016; Kong and Zweifel, 2021), and that a hierarchical organization would prioritize innate over learned negative valence (Isosaka et al., 2015; Kong and Zweifel, 2021). However, central amygdala circuits seem to be critical in the expressions of both of them, although by different mechanisms (Kong and Zweifel, 2021). On the basis of the known literature, we hypothesized a significant involvement of early visual cortices and amygdala, in addition to face- and word-specific processing areas, and we used DCM together with Bayesian model selection (Parametric Empirical Bayes for DCM, PEB; Zeidman et al., 2019a) to investigate the directional connectivity structure among these regions.

## 2. Materials and methods

### 2.1. Participants

Thirty-six volunteers (21 women; mean age ± SD: 28.0 ± 5.08; range: 18–38) took part in the fMRI study. Inclusion criteria were: being native speakers of the Italian language, right-handedness as assessed by the Edinburgh Inventory (Oldfield, 1971), no history of psychiatric or neurological disease, and no current use of psychoactive medications. The sample size was chosen according to published guidelines for fMRI experiments in healthy volunteers (Friston, 2012). The study was conducted according to the 2013 version of the Declaration of Helsinki and had been approved by the local Ethics Committee (protocol number: CE 134/2014/SPER/AOUMO), with all subjects giving their written informed consent before taking part in the study. Since one participant’s data were discarded due to excessive movement during the MR scanning session, the final sample for all the analyses included 35 subjects.

### 2.2. Stimuli

Faces and words were presented on an MRI-compatible display. Faces included photographs of happy (i.e., positive-valenced, *n* = 36), angry (i.e., negative-valenced, *n* = 36), and neutral (*n* = 36) facial expressions performed by 18 female and 18 male models (Radke et al., 2017). In order to reach the required number of trials, which was greater than the number of unique photographs, 12 faces of each category were presented twice (the stimuli to be repeated were randomly selected for each subject). Hair and non-facial contours were masked out in order to minimize their influences, and pictures were balanced for brightness and contrast values. They were presented in black and white on a black background to prevent color from influencing participants’ responses. Linguistic stimuli included positive (*n* = 18), negative (*n* = 18), and neutral (*n* = 18) words. Words were selected from the Italian version of the ANEW database (Affective Norms for English Words; Montefinese et al., 2014). As they were carefully balanced for the main distributional, psycholinguistic, and affective features known to affect the time it takes to encode a word, the number of suitable stimuli was limited, therefore 12 items of each category were presented twice, and 6 were presented three times, in order to match the number of face stimuli. Which items were presented two or three times was randomly defined within each subject. More specifically, words were balanced for length in letters, frequency, imageability, and concreteness. Positive and negative words were also balanced for squared valence and arousal (Table 1). Linguistic stimuli were presented in white lowercase Arial font on a black background.

**Table 1:** Descriptive statistics (mean ± SD) of length in letters, frequency of use, imageability, and concreteness, for positive, negative, and neutral words, and of valence and arousal for positive and negative words.

	Positive words	Negative words	Neutral words	*F*(2,48)	*t*(34)	*p* value
Length	7.22 (±2.39)	6.89 (±2)	7.06 (±1.89)	0.11	–	0.89
Frequency	19,305.89 (±25,084.41)	11,578.56 (±30,717.37)	34,431.78 (±69,249.36)	1.15	–	0.32
Imageability	7.49 (±0.95)	6.93 (±0.94)	7.46 (±1.40)	1.46	–	0.24
Concreteness	6.25 (±1.69)	6.46 (±1.19)	7.29 (±1.73)	2.24	–	0.12
Valence	8.28 (±0.25)	1.82 (±0.23)	–	–	−1.27^§^	0.21^§^
Arousal	6.32 (±0.74)	6.63 (±0.75)	–	–	1.26	0.22

F- *t*- and *p*-value for testing differences of the means are reported in the last three columns. ^§^For valence, *t*- and *p*-values refer to a test performed on the squared deviations of the valence scores from the center (i.e., 5) of the scale. *t*-values are reported for valence and arousal, while for the other features *F*-values are reported.

### 2.3. Procedure

**Figure 1:**
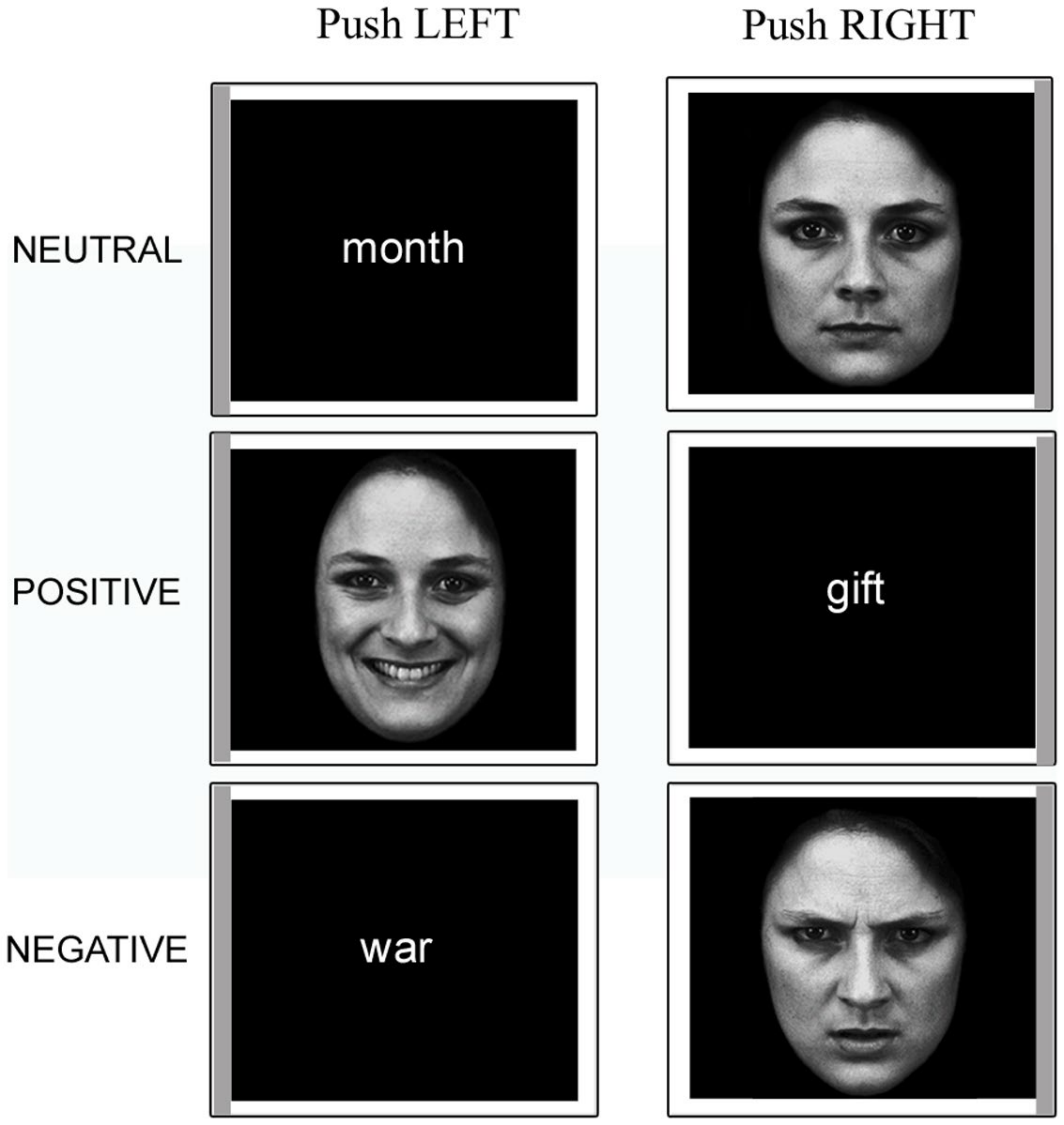
Example stimuli presented in the experimental task. Note the gray sidebars that indicate the required response. The faces have been adapted with permission from the Karolinska Directed Emotional Faces (KDEF; images IDs: F01NES, F01HAS, F01ANS; Lundqvist et al., 1998).

An event-related fMRI paradigm was employed. Participants read the task instructions before entering the scanner. Once inside, a two-button response pad was fixed under their right hand. They were asked to keep their gaze fixed on the center of the screen throughout the experiment. Each trial started with a white fixation cross on a black background followed by a stimulus (face or word). Each stimulus was presented within a thin white frame whose left or right side was of a darker (gray) shade and remained on the screen for 2 s (see Figure 1). Participants had to push the left button with their index finger or the right button with their middle finger if the gray sidebar appeared to the left or to the right of the stimulus, respectively. The inter-stimulus interval ranged pseudo-randomly between 2 and 18.8 s. Each participant performed four functional imaging runs; each run consisted of 72 trials, of which 36 faces (12 happy, 12 angry, 12 neutral) and 36 words (12 positive, 12 negative, 12 neutral). The AFNI (Cox, 1996; Cox and Hyde, 1997) *make_random_timing.py* function^1^ was used to simulate a series of randomized timing sequences for the trials of each stimulus category, from which the sequence with the best statistical power for the effects of interest was then identified with the *3dDeconvolve* program.^2^ Within each category, stimuli were presented in a pseudo-random order, with the constraint that no more than three consecutive stimuli belonging to the same class could occur. Two passive rest blocks were included at the beginning and at the end of each session (range 22.1–24.3 s). Each functional run lasted 8 min and the MRI session included 4 of them.

Participants performed a few practice trials inside the scanner before the experiment started. E-Prime 3.0 software (Psychology Software Tools, Pittsburgh, PA) was used to present the stimuli *via* the ESys functional MRI System^3^ remote display, and to collect behavioral responses.

At the end of the experiment, and outside the scanner, participants were asked to rate all the experimental stimuli for their valence and arousal on the Self-Assessment Manikin scale (SAM) (Bradley and Lang, 1994). The two rating questionnaires were delivered and completed on an Excel spreadsheet displayed on a tablet.

### 2.4. Behavioral data analyses

The average reaction times (RT) for the four conditions of interest (positive and negative faces, positive and negative words) were calculated for each volunteer, and the effect of valence within stimulus type (Faces, Words) was assessed with Wilcoxon signed-rank tests because of data non-normality. The average post-scanning ratings of Valence and Arousal for positive and negative faces and words were compared using paired *t*-tests.

### 2.5. fMRI data acquisition and preprocessing

MRI data were acquired with a GE SIGNA Architect 3.0 T MRI scanner and, for each volunteer, included 4 functional runs (gradient-echo echo-planar sequence, 46 axial slices, TR = 1,500 ms, voxel size: 3 × 3 × 2.7 mm with a 0.3 mm gap), and a high-resolution T1-weighted anatomical image (344 sagittal slices, TR = 2184.9 ms, TE = 3.09 ms, voxel size = 1 × 1 × 1 mm).

fMRI data were preprocessed using MATLAB version R2021a (The MathWorks Inc., Natick, Mass) and SPM12 (Wellcome Department of Imaging Neuroscience, London, United Kingdom). Functional volumes were slice-time corrected and realigned to the first volume acquired. The T1-weighted image was coregistered to the mean functional image and segmented using standard SPM tissue probability maps. The estimated deformation fields warp parameters from subject to MNI (Montreal Neurologic Institute) space were used to normalize the functional volumes to the MNI template implemented in SPM12. Finally, the functional data were smoothed with a 6x6x6 mm FWHM Gaussian kernel.

### 2.6. First- and second-level General Linear Model analyses of fMRI data

First-level (single-subject) analyses were performed as a multiple linear regression with six explanatory variables corresponding to the four stimulus classes of interest: positive faces (*Faces.pos*), negative faces (*Faces.neg*), positive words (*Words.pos*), negative words (*Words.neg*), neutral faces, and neutral words. Each condition was modeled by convolving the stimulus onset vectors for the corresponding stimulus class with a canonical hemodynamic response function (HRF), thus providing regressors of interest for the linear model (one for each class of stimuli) to be fitted to the fMRI data. The six parameters estimated by the motion correction algorithm were used as confound regressors. Linear contrasts were used to compare condition-specific effects and to obtain contrast images to be subsequently entered in a second-level (random-effects) group analysis. The contrasts of interest were: (a) [*Faces vs Rest*]; (b) [*Words vs Rest*]; (c) [*Faces vs Words*]; (d) positive vs. negative [*pos vs neg*]; (e) Faces-positive *vs* Faces-negative [*Faces.pos vs Faces.neg*], (f) Words-positive *vs* Words-negative [*Words.pos vs Words.neg*]. Additionally, an omnibus F-contrast was computed to test against the null hypothesis of the absence of any effect of interest. In the group analysis, gender was entered as a covariate.

SPM cluster-extent-based thresholding (with voxel-wise *p* < 0.001) was chosen to yield a whole-brain family-wise significance level *α* < 0.05 for the group-level statistical maps (Woo et al., 2014). Average beta values for each regressor of interest were extracted from different regions of interest (ROIs) using MarsBar version 0.45^4^ and plotted for detailed examination.

In order to identify the brain regions showing a valence effect for both faces and words, the intersection of the thresholded and binarized maps for the contrasts [*Faces.pos > Faces.neg*] and [*Words.pos > Words.neg*] was also computed, following the “Minimum Statistics compared to the Conjunction Null” approach described in Nichols et al. (2005).

### 2.7. Dynamic causal modeling

Dynamic causal modeling was performed using SPM12. This is a Bayesian framework to infer directed (effective) connectivity between brain regions (Friston et al., 2003). Since the purpose of the present study was to investigate the neural circuits differentially coding for positive and negative valence in the implicit processing of facial expressions and words, two different DCMs were performed, one for faces (Face-DCM) and one for words (Word-DCM).

#### 2.7.1. Volumes of interest selection and time series extraction

The starting point for a DCM analysis is the selection of a set of regions and their putative connections. The specific coordinates for each region were informed by the group-level GLM results and the known neuroimaging literature of emotional processing.

Namely, for the Face-DCM, five volumes of interest (VOIs) were chosen based on the group activation *t*-map peaks as follows. The areas more specifically involved in face processing, i.e., right Fusiform Face Area (FFA) and right Middle Frontal Gyrus (MFG), were identified *via* the [*Faces > Words*] contrast. The regions involved in emotional valence were selected *via* the [*pos > neg*] contrast (bilateral secondary visual area, V2), and the [*Faces > Rest*] contrast (bilateral primary visual area, V1, and right amygdala).

The selection of regions for the Word-DCM—five VOIs as well—was performed with a similar strategy. The left Visual Word Form Area (VWFA) and left Inferior Frontal Gyrus (IFG) were identified *via* the [*Words > Faces*] contrast, the bilateral V2 *via* the [*pos > neg*] contrast, and the bilateral V1 *via* the [*Words* > *Rest*] contrast. Although the left amygdala was not part of the activated regions for the [*Words > Rest*] contrast, when using a corrected statistical threshold for the whole brain, we included it as a node for the Word-DCM (using symmetrical coordinates to the right amygdala VOI), because of the extant evidence of its involvement in processing of emotional words (Hamann and Mao, 2002; Kensinger and Schacter, 2006; Lewis et al., 2007; Nakamura et al., 2020).

These VOIs were fitted to the individual subjects according to the following automated procedure. For each subject and each VOI, a subject-specific peak for the same contrast was identified within 9 mm from the respective group-level cluster peak. Then, for each of these subject-specific peaks, the first eigenvariate time series was extracted from the preprocessed functional data from a 5 mm-radius sphere centered around it, adjusted for the effects of interest (i.e., regressing out the effects of no interest). All the voxels within this sphere were used in the computation of the first eigenvariate, with no threshold applied. It should be noted that the coordinates initially identified *via* the group contrasts for each VOI were used only as “starting point” for the procedure of subject-wise VOI specification; thus, for every subject and every VOI, the procedure successfully identified a significant local peak within 9 mm from the starting coordinates. The Euclidean distances between the center of the subject-level spheres and the initial group-level peaks were on average 2.55 and 3.28 mm for Face- and Word-DCM, respectively. More details about time series extraction are provided in Table 1, Supplementary material.

#### 2.7.2. Model specification

**Figure 2:**
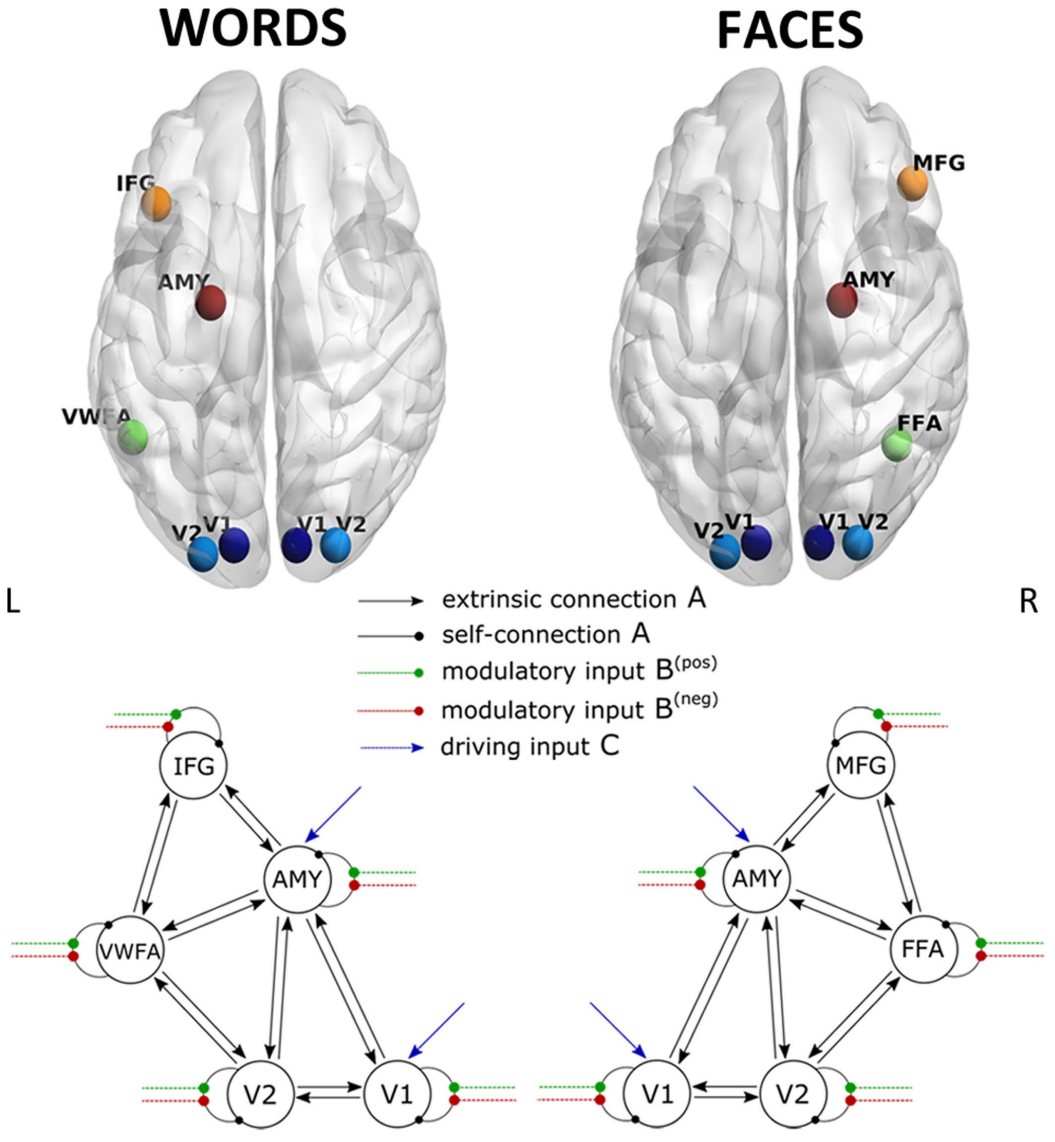
Visualization of the VOIs (top) and of the full models (down) selected for Word- (left) and Face- (right) DCM. Render visualized using BrainNet Viewer (Xia et al., 2013). V1, primary visual area; V2, secondary visual area; IFG, inferior frontal gyrus; AMY, amygdala; VWFA, visual word form area; MFG, middle frontal gyrus; FFA, fusiform face area.

The DCM model space is established by specifying a general connectivity structure to the set of included VOIs. Two one-state bilinear DCMs (Friston et al., 2003) were implemented, one for faces and one for words. Three types of parameters were estimated: (1) endogenous parameters, measuring the average effective connectivity across experimental conditions (specified by the **A**-matrix); (2) modulatory parameters, reflecting changes in effective connectivity due to experimental conditions (specified by the **B** 3D-matrix); (3) driving parameters, showing how the single brain regions respond to experimental stimuli (specified by the **C** matrix). Concerning the specification of extrinsic (i.e., between-region) connectivity, bidirectional connections were enabled in our model between V1 and V2, V2 and FFA, FFA, and MFG for faces, and between V1 and V2, V2 and VWFA, VWFA, and IFG for words; in addition, bidirectional connections were enabled between the amygdala and any other VOI, for both Face-DCM and Word-DCM (Figure 2). Moreover, each brain region was equipped with an inhibitory self-connection, specified by the elements on the leading diagonal of the **A**-matrix, modeling the region’s excitatory-inhibitory balance (Bastos et al., 2012).

For Face-DCM as well as for Word-DCM, both faces and words were set as driving input to V1 and the amygdala. Even though the amygdala’s activation was not significantly different between positive and negative stimuli in the group analysis, this VOI was included as a target for driving input because it belongs to the subcortical visual pathway and is known to participate in the recognition of emotional signals (Adolphs, 2002).

Finally, the modulatory effect of valence for faces or words was enabled on the self-connections of each region of the two models, respectively. As recommended by Zeidman et al. (2019a), restricting the modulatory effects to self-connections improves parameter identifiability. Since self-connections are constrained to be negative (i.e., inhibitory), both endogenous and modulatory self-connection parameters are unitless log-scaling parameters that multiply up or down the default value of −0.5 Hz. As a consequence, the more positive is the self-connection parameter, the more inhibited is the region.

Therefore, four conditions were defined for each DCM: (i) positive faces, negative faces, faces, and words for Face-DCM, (ii) positive words, negative words, faces, and words for Word-DCM. For each condition, “input” vectors representing stimulus timing were mean-centered to improve the model evidence and give the matrix **A** a simpler interpretation (the average connectivity; Zeidman et al., 2019a).

DCM estimation was performed using the function *spm_dcm_fit*, which fits DCM to the data using Variational Laplace. We will refer to the model just specified as the “full” model, since all parameters of interest were therein switched on.

#### 2.7.3. Parametric empirical Bayes

In DCM, a “first-level” estimation of each subject’s connectivity parameters is typically followed by a “second-level” quantification of the commonalities and differences across subjects. The second-level analysis was performed using the method of Parametric Empirical Bayes (PEB) (Friston et al., 2016). The PEB method rests on having defined and estimated only one “full” DCM at the single-subject level, where all possible connections of interest are present. Other candidate models are specified simply by choosing which parameters should be “switched on” and “switched off.” More specifically, instead of manually defining a choice set of reduced models, an automatic search was performed using the SPM function *spm_dcm_peb_bmc*. The automatic PEB was implemented separately for matrices **A** and **B** (Zeidman et al., 2019b). For both faces and words, fixed connections were described by 38 s-level parameters: 2 between-subjects effects (group mean and gender) times 19 DCM parameters (5 self-connections, 14 between regions connections). On the other hand, modulatory inputs were described by 20 parameters: 2 between-subjects effects, times 10 DCM parameters (5 for each valence type). Gender and age were used as covariates to account for a potential biasing effect of these variables on the estimation of group mean parameters. The final step of the group analysis is a Bayesian Model Averaging (BMA) procedure, which identifies and reports the parameters with an estimated posterior probability of being nonzero greater than 0.95 (see Supplementary Materials for details).

## 3. Results

### 3.1. Behavioral data and questionnaires

**Figure 3:**
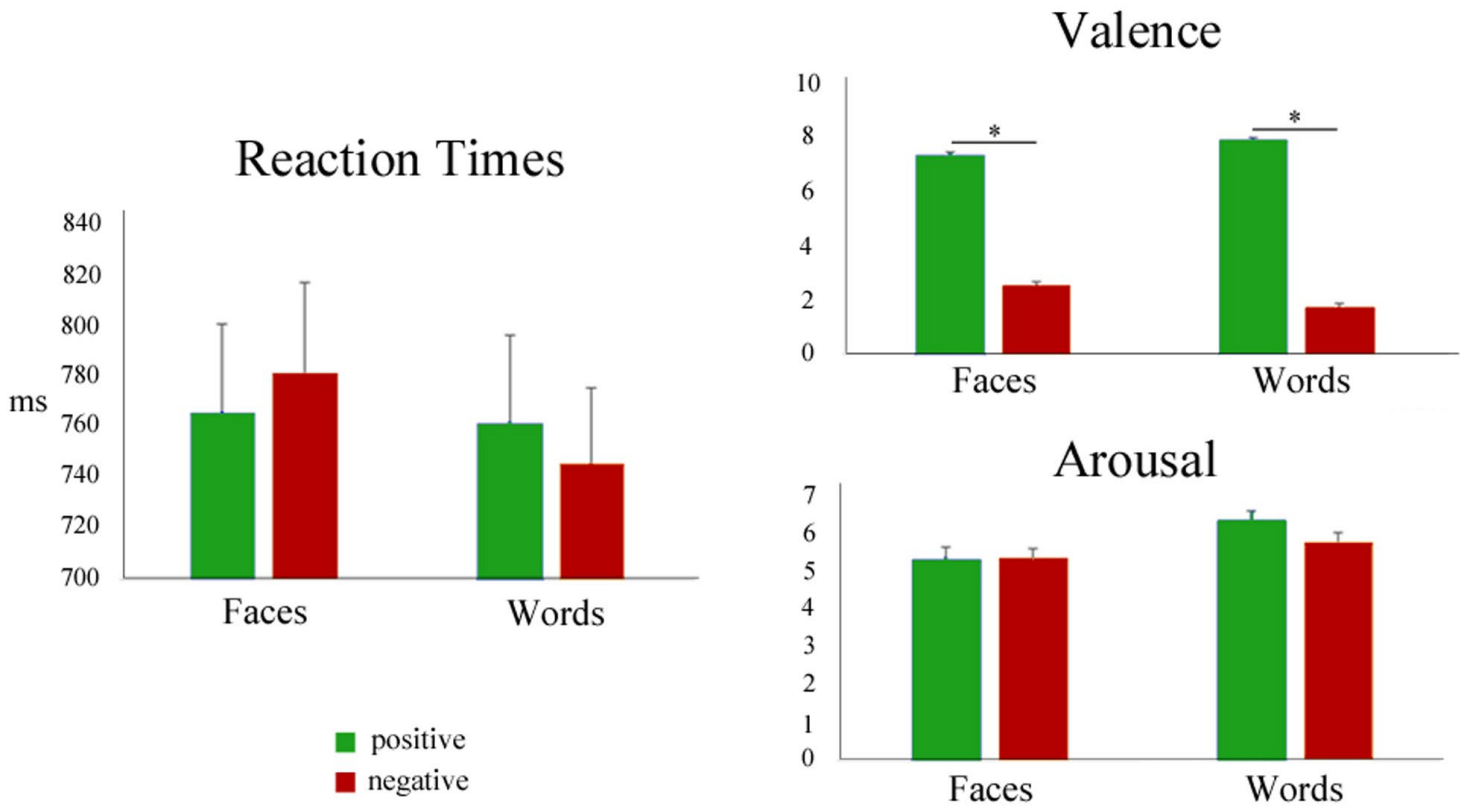
Left: Mean reaction times during positive (green) and negative (red) faces and words. Right: mean post-scanning ratings for valence and arousal.

The mean reaction times and post-scanning ratings of all the experimental stimuli are summarized in Figure 3 and Table 2. Since the focus of the present study was the comparison of positive and negative emotional valence of faces and words, the mean RT and post-scanning ratings for neutral stimuli are reported in Table 2 for the sake of completeness, but were not further analyzed.

**Table 2:** Average post-scanning ratings of valence and arousal (range 1–9) and response times (ms) for positive, negative, and neutral stimuli.

	FACES average ratings (SD)			WORDS average ratings (SD)		
	Positive	Negative	Neutral	Positive	Negative	Neutral
Valence	7.3 (0.9)	2.5 (0.7)	4.6 (0.6)	7.9 (0.7)	1.7 (0.5)	5.0 (0.3)
Arousal	5.3 (1.5)	5.3 (1.8)	2.4 (1.3)	6.3 (1.6)	5.7 (1.5)	2.1 (1.4)
Reaction times	765.3 (210.6)	781.4 (211.9)	770.7 (202.4)	761.3 (210.0)	745.7 (177.7)	742.7 (205.3)

SD, standard deviation.

Differences in RT means related to valence were not significant for Faces or Words, as assessed by Wilcoxon signed-rank tests [*Faces.neg vs Faces.pos*], (*p* = 0.10; *[Words.neg vs Words.pos]*, *p* = 0.13), showing that positive and negative stimuli had similar processing demands. Paired *t*-tests comparing the arousal ratings for negative and positive stimuli (faces and words, separately) were also nonsignificant, confirming that positive and negative stimuli were well-matched in this dimension (Faces: *t*(34) = 0.045, *p* = 0.96; Words: *t*(34) = −1.68, *p* = 0.10). As expected, paired *t*-tests for the valence ratings showed highly significant differences between negative and positive stimuli, for both faces and words (Faces: *t*(34) = −20.18, *p* < 0.001; Words: *t*(34) = −34.25, *p* < 0.001), thus validating the employed category grouping of the experimental stimuli. When analyzing the squared deviations of the valence scores from the center of the scale (i.e., 5), a paired *t*-test showed no significant difference between negative and positive faces (*t*(34) = 0.86, *p =* 0.40), but significantly higher values for negative words compared to positive ones (*t*(34) = 3.70, *p <* 0.001).

### 3.2. GLM analyses results

#### 3.2.1. Effect of stimulus type

**Figure 4:**
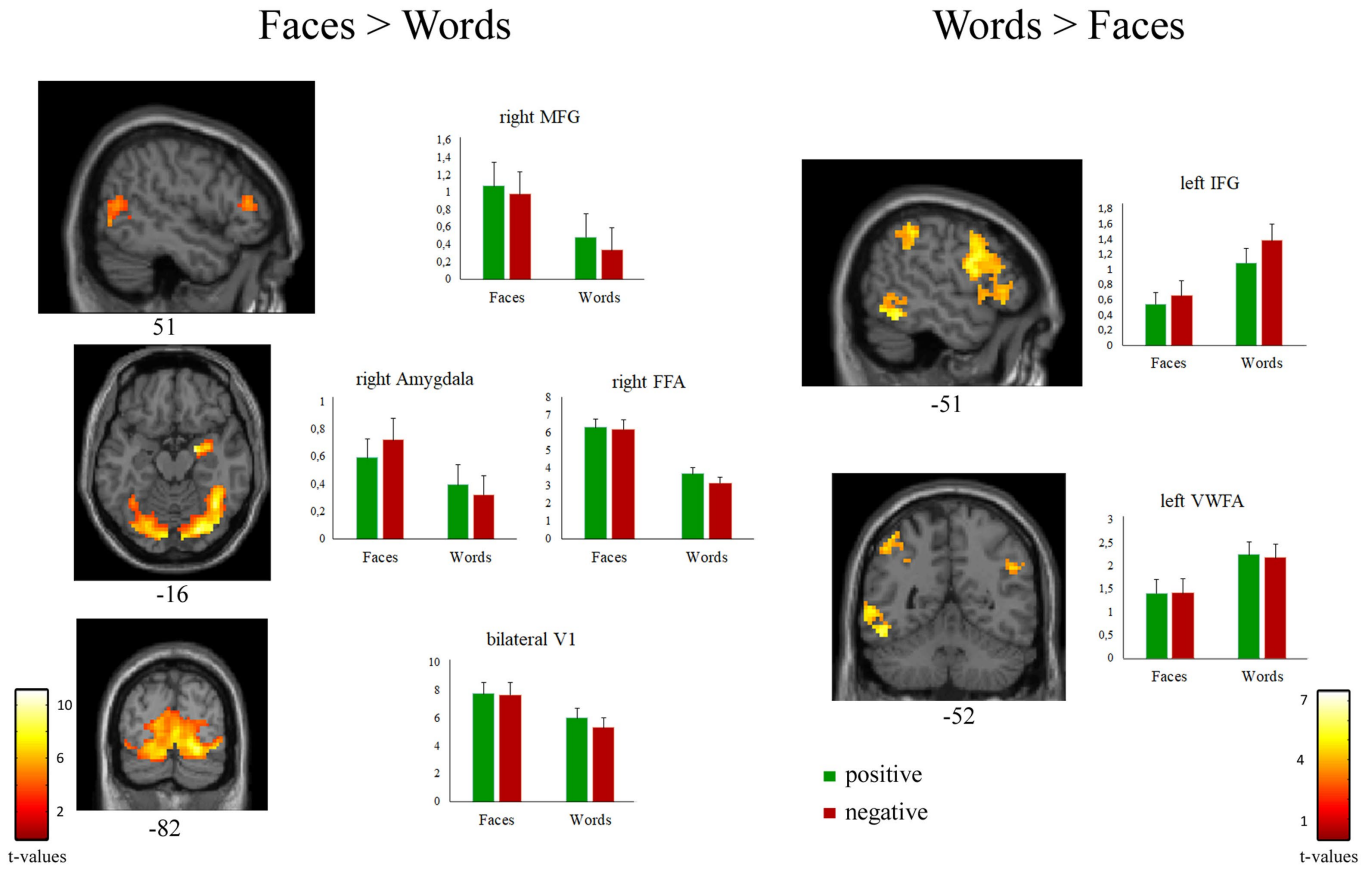
Suprathreshold clusters of activation for the contrasts [*Faces > Words*] (left) and [*Words > Faces*] (right). Bar plots represent the cluster-averaged estimates of GLM coefficients (beta values) for each regressor of interest.

As expected, the comparison [*Faces > Words*] revealed significant activations of regions belonging to the “core face network,” such as bilateral occipital face area (OFA), right fusiform face area (FFA), and superior temporal sulcus. A significant activation of bilateral thalamus and of the extended face network, including right amygdala and right middle frontal gyrus, was also observed (Figure 4 left; Table 3A).

**Table 3:** Peak coordinates of the contrasts [*Faces > Words*] (cluster size threshold *K* ≥ 35 voxels) and [*Words > Faces*] (*K* ≥ 41, corrected at *α* < 0.05).

Anatomical location	BA	Side	Cluster size (voxels)	Peak z-score	MNI coordinates (x, y, z)		
*(A) Faces > Words*							
Fusiform gyrus (FFA), occipital fusiform gyrus (OFA), calcarine cortex, lingual gyrus, superior temporal sulcus	37, 19, 18, 17	l/*r*	2,617	7.08	42	−55	−19
Amygdala		*r*	90	6.62	21	−7	−16
Thalamus		*r*	112	6.20	21	−31	−1
Middle frontal gyrus (dlPFC)	9	*r*	47	4.57	51	35	14
Thalamus		l	35	4.31	−27	−25	−7
*(B) Words > Faces*							
Inferior occipital gyrus	17	l	41	5.63	−24	−97	−10
Superior parietal lobule, supramarginal gyrus, angular gyrus	39, 40	l	403	4.90	−30	−64	50
Inferior (VWFA), middle, and superior temporal gyri	21, 37	l	245	4.81	−66	−43	2
Inferior frontal gyrus (pars opercularis, pars triangularis)	44, 45	l	535	4.73	−51	5	26
Angular gyrus	39	*r*	54	3.92	48	−52	32

*r*, right; *l*, left; BA, Brodmann area.

The contrast [*Words > Faces*] identified a network of left-lateralized linguistic regions, including the inferior frontal gyrus (pars opercularis and triangularis), the supramarginal gyrus, and the visual word form area of the fusiform gyrus. The left inferior frontal gyrus and the bilateral angular gyrus were also significantly activated (Figure 4 right; Table 3B).

#### 3.2.2. Effect of valence

**Figure 5:**
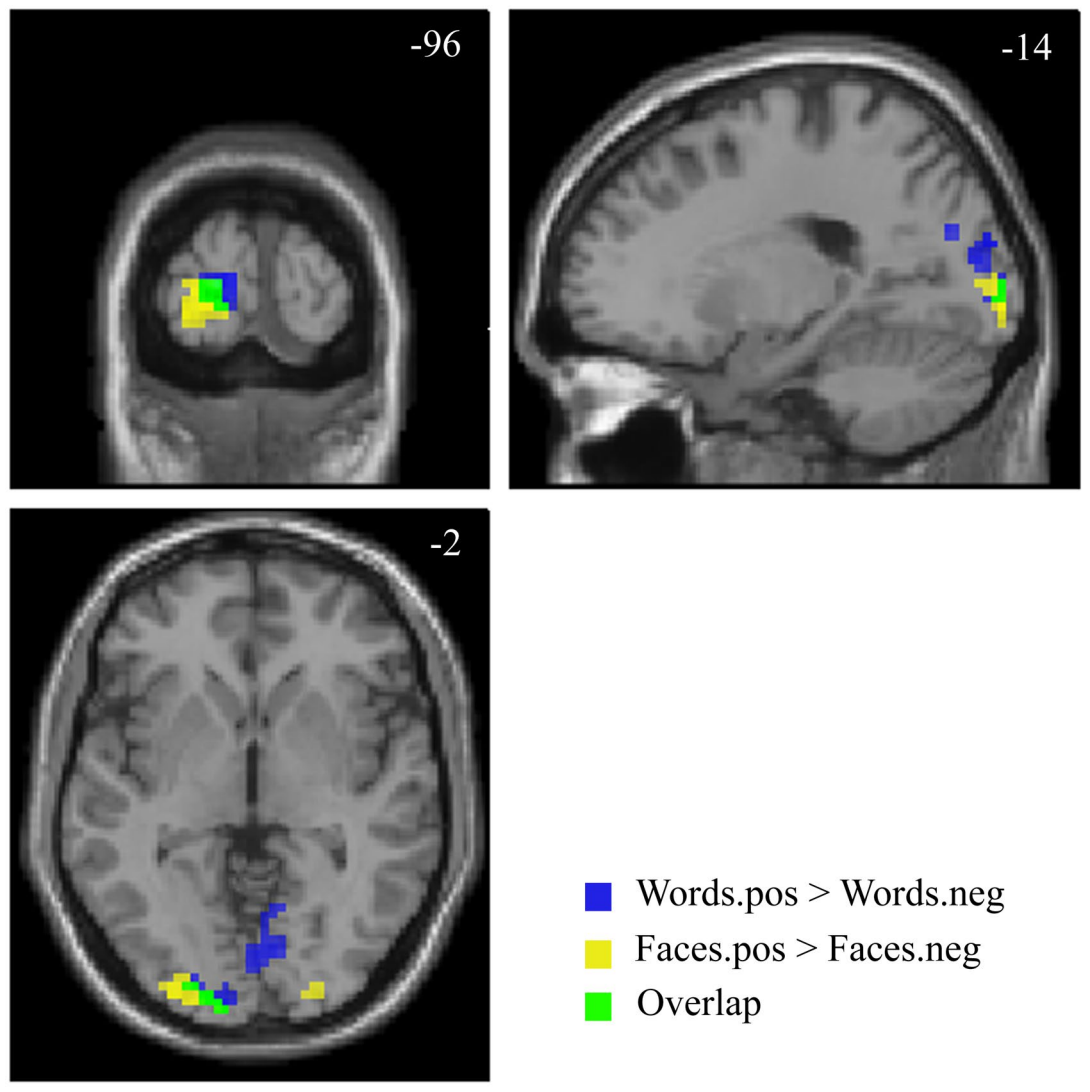
Suprathreshold clusters for the contrasts [*Faces.pos > Faces.neg*] (yellow) and [*Words.pos > Words.neg*] (blue), with their overlap, representing the areas showing a significant effect of valence for both faces and words, depicted in green.

The contrast [*Faces.pos > Faces.neg*] revealed two clusters in the extrastriate visual cortex (V2, BA 18–19; Figure 5 and Table 4A), with both classes of stimuli showing positive activation with respect to the resting baseline.

**Table 4:** Peak coordinates of the contrast [*Faces.pos > Faces.neg*] (A) and [*Words.pos > Words.neg*] (B).

Anatomical location	BA	Side	Cluster size (voxels)	Peak z-score	MNI coordinates (x, y, z)		
*(A) Faces*							
Inferior occipital gyrus	19	l	100	4.70	−27	−94	5
Inferior and superior occipital gyri	19	*r*	68	4.36	27	−88	8
*(B) Words*							
Fusiform gyrus, bilateral lingual gyrus, calcarine cortex, cuneus	17, 18, 19	*r*	746	4.97	27	−76	−13

Cluster-extent-based thresholding at *α* < 0.05, with single-voxel *p* < 0.001. *r*, right; l, left; BA, Brodmann area.

The corresponding contrast for words [*Words.pos > Words.neg*] identified a wider occipital network of activated clusters in the bilateral lingual and fusiform gyri, cuneus, and calcarine cortex (Figure 5 and Table 4B). No regions of increased signal for negative stimuli compared to positive ones were detected, either for faces or words.

The areas showing a significant effect of valence for both faces and words were limited to the bilateral secondary visual cortex, with a larger extent on the left side (Figure 5).

### 3.3. Dynamic causal modeling results

The *spm_dcm_fmri_check* function was used to calculate the percentage of explained variance in order to evaluate the performance of model inversion. Across all included subjects, an average explained variance of 28.4% (SD 11.4%) for faces and of 21.2% (SD 11.2%) for words was obtained.

#### 3.3.1. Face-associated connectivity

**Figure 6:**
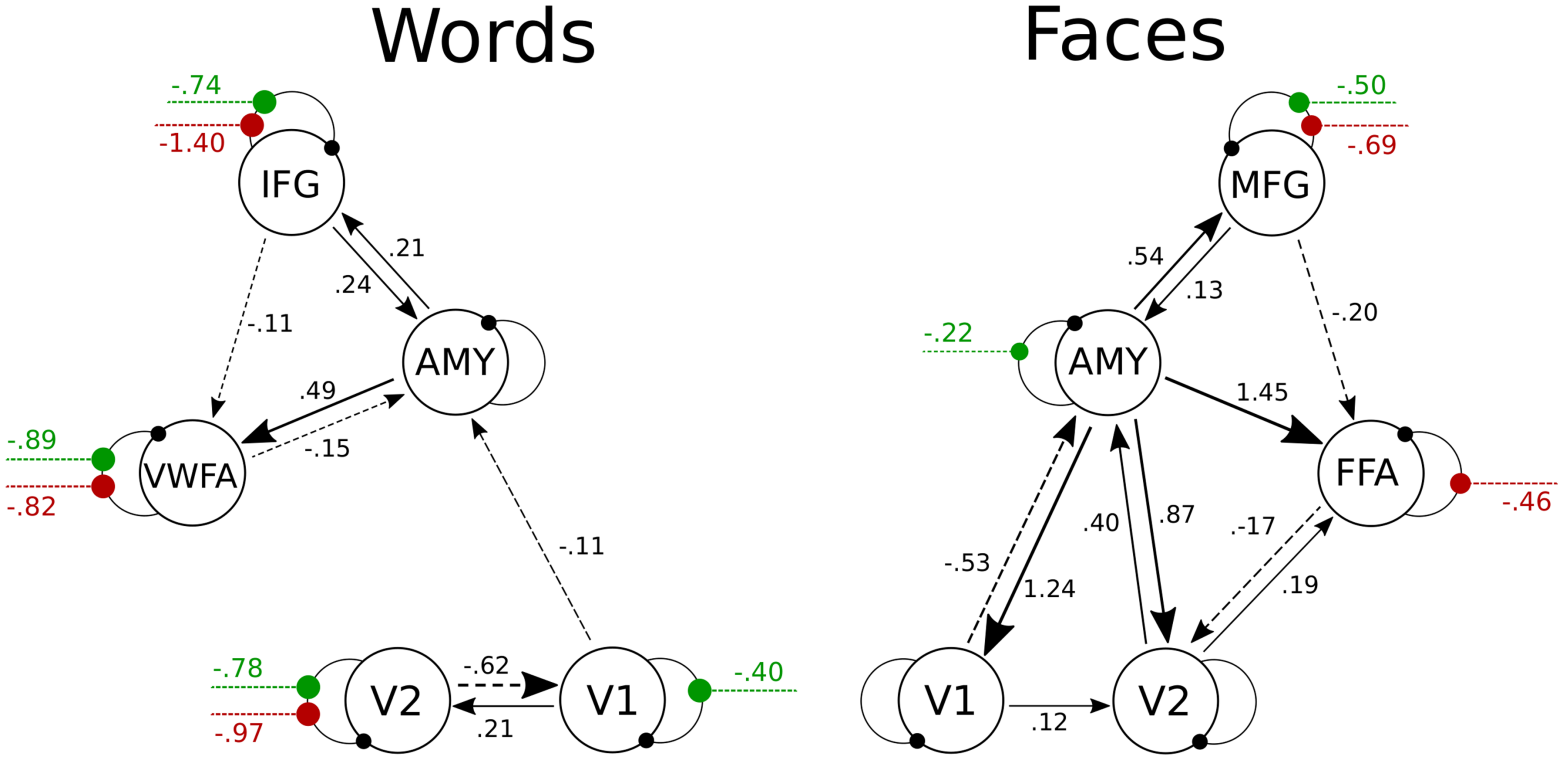
Visualization of PEB results for the Face- and the Word-DCMs. Only parameters with 95% probability of being nonzero are displayed. To avoid visual clutter, values of endogenous self-connections are not shown, but are included in Table 5. Between-region connectivity parameters are in units of Hz; negative numbers (dotted lines) indicate inhibition. Green and red lines represent the modulations on self-connections by positive and negative stimuli, respectively; these parameters are unitless. More details about the posterior parameter estimates for the **A** and **B** matrices are given in Supplementary Table S2 and Supplementary Figure S1. V1, primary visual area; V2, secondary visual area; IFG, inferior frontal gyrus; AMY, amygdala; VWFA, visual word form area; MFG, middle frontal gyrus; FFA, fusiform face area.

The BMA was calculated over 256 models for fixed connections (**A**-matrix) and 109 for modulatory inputs (**B**-matrix). For the group mean parameters, the presence of a strong endogenous self-inhibition was observed in V1, whereas the amygdala’s endogenous self-inhibition was weak. The coupling parameters revealed excitatory connections from the amygdala to V1, V2, FFA, and MFG. As can be gleaned by the connectivity scheme in Figure 6, the strength of these connections was further increased for positive faces, as a consequence of the modulation of the amygdala’s self-connection. An inhibitory connection was found from FFA to V2; its inhibitory strength was further increased during the processing of negative faces *via* their modulatory input on the FFA’s self-connection. Both positive and negative faces modulated the self-connection of the MFG, increasing overall its activation. Further details about self-connections parameters and their modulations are shown in Table 5A.

**Table 5:** Estimated self-connections parameters obtained using PEB.

	Region	Endogenous connectivity	Modulation of positive stimuli	Modulation of negative stimuli
*(A) Faces*	V1	1.23 (0.05)	–	–
	V2	–	–	–
	FFA	0.10 (0.04)	–	−0.46 (0.07)
	MFG	–	−0.50 (0.10)	−0.69 (0.11)
	AMY	−0.83 (0.04)	−0.22 (0.07)	–
*(B) Words*	V1	0.90 (0.06)	−0.40 (0.08)	–
	V2	0.21 (0.03)	−0.78 (0.09)	−0.97 (0.10)
	VWFA	–	−0.89 (0.13)	−0.82 (0.13)
	IFG	–	−0.74 (0.17)	−1.40 (0.16)
	AMY	−0.58 (0.04)	–	–

The second column contains the average connectivity strength (SD in parenthesis) across experimental conditions (diagonal of **A**-matrix), whereas the last two columns refer to the modulatory influence of positive and negative stimuli. Variables in the Table are the log of scaling parameters that multiply up or down the default self-connection value of-0.5 Hz. For a graphical interpretation of the parameter estimates, see also Supplementary Figure S1. V1, primary visual area; V2, secondary visual area; IFG, inferior frontal gyrus; AMY, amygdala; VWFA, visual word form area; MFG, middle frontal gyrus; FFA, fusiform face area. *Empty cells indicate parameters with probability of being nonzero lower than 0.95. These parameters are not considered meaningful.

#### 3.3.2. Word-associated connectivity

In the case of the Word-DCM, BMA was calculated over 179 models for the **A**-matrix and 106 for the **B**-matrix. The strong self-inhibition of V1 and the weak self-inhibition of the amygdala were similar to what was found in Face-DCM. In addition, there were excitatory connections from the amygdala to IFG and VWFA. A significant excitatory connection from V1 to V2 was observed; its strength was further increased during the processing of positive faces *via* their modulatory input on the V1’s self-connection. Self-connections of V2, VWFA, and IFG were modulated by both positive and negative words; all of these modulations were negative, indicating a stronger activation of the mentioned regions in presence of both stimuli. Further details are provided in Figure 6 and Table 5B.

## 4. Discussion

The main purpose of the present study was to investigate similarities and differences in neural activations coding for valenced facial expressions and words, using an emotionally-implicit visual processing task. We found evidence for a differential response to negative and positive valence across both faces and words only in early visual cortices (mainly V2). DCM analyses revealed that, for faces, this effect was chiefly mediated by a facilitation of amygdalar activity by positive stimuli and of FFA activity by negative ones; for words, the effect was mainly imputable to a facilitation of V1 activity by positive stimuli.

Recent perspectives hypothesize the existence of a network of regions coding for valence without a regional specificity for positive and negative stimuli (Chikazoe et al., 2014). The notion is supported by a recent meta-analysis arguing for a flexible affective workspace, processing both positive and negative affect (Lindquist et al., 2016). This valence-general neural space includes anterior insula, rostral and dorsal ACC, ventromedial prefrontal cortex, amygdala, ventral striatum, thalamus, and occipitotemporal cortex. Recently, Miskovic and Anderson (2018) suggested that this general valence coding works together with differentiated modality-specific representations of valence, with a major role of the sensory cortices. Supporting this hypothesis, a recent work using multivariate pattern analysis showed that the specific valence of visual and auditory stimuli can be successfully decoded by the activity of the respective sensory cortices (Shinkareva et al., 2014). A meta-analytic study by Satpute et al. (2015) also demonstrated the involvement of early sensory areas in constructing modality-specific affective experience for all sensory modalities.

Our study showed a differential activation of extrastriate visual cortex for positive and negative stimuli, for both faces and words, confirming previous findings suggesting the importance of early sensory brain cortices for affective experience and valence decoding (Satpute et al., 2015; Miskovic and Anderson, 2018). Even though for emotion-related word processing the extant experimental evidence is a bit more controversial (Kissler et al., 2006; Citron, 2012; Reisch et al., 2020), the present results speak to the importance of the early perceptual stages in valence processing, not only for facial expressions, but also for words. The involvement of early visual cortices during emotional word processing has been in fact reported in a number of fMRI studies. Herbert et al. (2009) identified a robust activation pattern in the left amygdala and in the left extrastriate visual cortex during the reading of pleasant adjectives (compared to unpleasant or neutral adjectives). A single cluster of increased signal in the left extrastriate cortex was reported during a word categorization task, compared to a picture categorization task (with emotional stimuli), by Flaisch et al. (2015). Finally, a functional coupling between the right extrastriate cortex and the right amygdala was found by Tabert et al. (2001) during unpleasant emotional word processing.

In the present study, we used Dynamic Causal Modeling to elucidate the mechanisms by which the activity of early sensory cortices is modulated by emotional valence of visual stimuli. In accordance with a recent review of functional and effective connectivity studies (Underwood et al., 2021), a dynamic and context-dependent interplay between cortical and subcortical regions was expected, and a key role of the amygdala was plausible for both faces and words. The main novelty of the present study is represented by the DCM results suggesting that different mechanisms may be at play in the extrastriate visual cortices in processing the affective valence of facial expressions and words: (i) V2 activation during emotional face processing seems to depend on excitatory inputs from the amygdala, which is itself modulated by positive stimuli, and on inhibitory inputs from FFA, which is modulated by negative faces; (ii) on the other hand, V2 activity during emotional word coding appears to rely more on excitatory inputs from V1, whose activity is modulated by positive stimuli.

Therefore, although some previous fMRI studies have reported a statistical association between the activations of the extrastriate cortex and the amygdala in both left and right hemispheres during affective word reading (Tabert et al., 2001; Herbert et al., 2009), our DCM findings do not provide evidence for a significant direct message passing between the amygdala and the secondary visual cortex during emotional words processing. However, a significant bidirectional coupling between the amygdala and the VWFA was observed in the present study, which suggests that the amygdala can detect the affective content of emotional words even before a detailed orthographic decoding, and may in turn influence the reading network (Nakamura et al., 2020).

In summary, this study supports the notion that the emotional valence of both faces and words can be discriminated in early sensory regions *via* two different mechanisms.

Mainly, the amygdala, as a key component of the subcortical visual route, is crucial for valence coding during the processing of faces, whereas for words, the direct thalamic pathway to primary visual cortex may play a stronger role in valence coding. Importantly, this does not necessarily imply that valence is discriminated early *in a temporal sense* in early visual cortices. As suggested by our results, the modulation may occur as a consequence of a top-down signal from hierarchically-higher visual areas (e.g., FFA and VWFA) and frontal regions (e.g., IFG and MFG).

While face and word stimuli differ in various perceptual aspects, the different circuits and patterns of directed connectivity identified in this study may also reflect differences in the innate or acquired capacity to respond to faces and words, respectively. For what concerns perception, the ability to interpret facial expressions of emotions has been shown to be present at a very young age (Nelson et al., 1979), and the production of emotional expressions is also often considered as innate and universal (Izard, 1994; Ekman, 2016; see however, more recent data suggesting a “theory of constructed emotion,” Feldman Barrett, 2017). It should also be noted that a generalization of our findings to every exemplar of such categories was beyond the scope of the present study (and likely addressable only with a meta-analysis approach).

## 5. Limitations of the study and further research

To our knowledge, this is the first effective connectivity study using PEB to compare putative neural circuits for faces and words, chosen here as typical instances of largely innate and largely acquired emotional stimuli, respectively; it provides novel insight into the role of early sensory cortices in such processes. However, we acknowledge a number of limitations that should be addressed in further experimental work.

First, our protocol involved only implicit emotional processing. While this was a design choice, it may be argued that a greater (or different) recruitment of neural circuits would be observed when processing of emotional information is explicitly required by the task; on the other hand, implicit task protocols are widely used in psychological and neurophysiological research, and they can be considered more ecological than the explicit tasks in most situations.

Second, emotional visual stimuli were limited to faces and only one type of facial expression was used for each valence class (i.e., happy for positive, angry for negative). How the current findings would change were other sets of visual stimuli employed — e.g., painful expressions, or IAPS stimuli (Lang et al., 2005) — remains to be determined.

Finally, words and faces are very different kinds of objects, therefore our stimuli do not differ only because their recognition is either primarily innate or culturally acquired. Future research could explore innate and acquired valence using stimuli with matched structural characteristics or, alternatively, more sophisticated paradigms where stimuli could be conditioned to modify their innate valence.

## Data availability statement

The raw data supporting the conclusions of this article will be made available by the authors, without undue reservation.

## Ethics statement

The studies involving human participants were reviewed and approved by Comitato Etico dell’Area Vasta Emilia Nord, Azienda Ospedaliero-Universitaria di Modena, Università di Modena e Reggio Emilia (Italy). The patients/participants provided their written informed consent to participate in this study. Written informed consent was obtained from the individual(s) for the publication of any identifiable images or data included in this article.

## Author contributions

DB, EB, FL, and GP contributed to the design of the experiment. DB, EB, and GP contributed to data collection. DB, RM, EB, and GP contributed to data analysis. All authors contributed to the interpretation of the results and manuscript writing.

## Funding

Funding for this study was awarded by the Ministero dell’Istruzione, dell’Università e della Ricerca (Project title: “The Good and the Bad of Sensory Experience: Understanding the Impact of Emotionally Charged Stimuli on Cognition and Behavior, and the Brain’s Mechanisms to Cope with Them;” Grant number: CUP E94I19000630005).

## Conflict of interest

The authors declare that the research was conducted in the absence of any commercial or financial relationships that could be construed as a potential conflict of interest.

## Publisher’s note

All claims expressed in this article are solely those of the authors and do not necessarily represent those of their affiliated organizations, or those of the publisher, the editors and the reviewers. Any product that may be evaluated in this article, or claim that may be made by its manufacturer, is not guaranteed or endorsed by the publisher.

## Footnotes

^1^ https://afni.nimh.nih.gov/pub/dist/doc/program_help/make_random_timing.py.html

^2^ https://afni.nimh.nih.gov/pub/dist/doc/htmldoc/programs/3dDeconvolve_sphx.html

^3^ http://www.invivocorp.com

^4^ http://marsbar.sourceforge.net/
