# Supplementary material for: Neural correlates of emotional valence for faces and words
Source: Front Psychol. 2023 Feb 23;14:1055054. doi: 10.3389/fpsyg.2023.1055054 (PMC9996044; doi:10.3389/fpsyg.2023.1055054)
Supplement: Supplementary file 1 [file Table_1.DOCX]

***Supplementary Material***

1. **Supplementary Data**

**Details of the group-level DCM procedure using PEB**

The search algorithm implemented by the SPM function *spm_dcm_peb_bmc* executes an iterative procedure to discard parameters that do not contribute to model evidence. Then, the model evidence and parameters for the reduced models obtained with all possible combinations of surviving parameters can be derived from the full one using Bayesian Model Reduction (BMR; Friston and Penny, 2011). Finally, a Bayesian Model Averaging (BMA; the average of the parameters weighted by the models’ posterior probabilities) was calculated over the candidate models in order to yield the parameter configuration of the final model.

1. **Supplementary Figures and Tables**

**Supplementary Table 1.** Details of VOIs extraction. For each VOI, the table shows (MNI) coordinates and contrasts used for the extraction of the time series, the average center of the local 5mm-sphere, and its distance to the group-level peak.

| **DCM type** | **VOI** | **Peak coordinates** | **Contrast** | **Average center of local sphere** | **Distance (mm)** |
| --- | --- | --- | --- | --- | --- |
| Face | l/r V1 | (-12, -88, 2)  (12, -88, 2) | Faces > Rest | (-11.0, -92.5, -0.57)   (11.1, -90.8, -0.1) | 4.35 |
| Face | r AMY | (21, -7, -16) | Faces > Rest | (20.9, -5.6, -15.8) | 1.46 |
| Face | r FFA | (42, -55, -19) | Faces > Words | (40.4, -54.3, -20.5) | 2.29 |
| Face | r MFG | (51, 35, 14) | Faces > Words | (50.7, 34.9, 13.6) | 0.52 |
| Face | l/r V2 | (-24, -91, 5)  (27, -88, 8) | pos > neg | (-23.9, -92.8, 3.7)  (27.1, -88.8, 7.7) | 4.10 |
| Word | l/r V1 | (-12, -88, 2)  (12, -88, 2) | Words > Rest | (-9.6, -90.6, 0.3)  (11.5, -89.1, -0.2) | 2.94 |
| Word | l AMY | (-21, -7, -16) | Words > Rest | (-22.9, -5.1, -14.1) | 3.31 |
| Word | l VWFA | (-51, -52, -13) | Words > Faces | (-49.6, -53.8, -12.4) | 2.34 |
| Word | l IFG | (-42, 26, -4) | Words > Faces | (-45.1, 24.8, -2.3) | 3.68 |
| Word | l/r V2 | (-24, -91, 5) (27, -88, 8) | pos > neg | (-23.9, -92.8, 3.7) (27.1, -88.8, 7.7) | 4.10 |

l = left; r = right. V1 = primary visual area; V2 = secondary visual area; IFG = inferior frontal gyrus; AMY = amygdala; VWFA = visual word form area; MFG = middle frontal gyrus; FFA = fusiform face area.

**Supplementary Table 2.** Mean and standard deviation of endogenous connections obtained using PEB. The table specifies the values of the group-mean intrinsic connectivity across experimental conditions (**A**-matrix) for faces (left) and words (right). Numbers in brackets are the standard deviations. Between-region connections are in units of Hz, whereas self-connections parameters are unitless logs of scaling parameters that multiply up or down -0.5 Hz. The first column indicates the parameter index in Supplementary Figure 1s.

| n |  | From region | To region | Value (SD) |  | From region | To region | Value (SD) |
| --- | --- | --- | --- | --- | --- | --- | --- | --- |
| 1 | a) Faces | V1 | V1 | 1.23 (0.05) | b) Words | V1 | V1 | 0.90 (0.06) |
| 2 |  | V1 | V2 | 0.12 (0.03) |  | V1 | V2 | 0.21 (0.02) |
| 3 |  | V1 | AMY | -0.53 (0.04) |  | V1 | AMY | -0.11 (0.03) |
| 4 |  | V2 | V1 | - |  | V2 | V1 | -0.62 (0.07) |
| 5 |  | V2 | V2 | - |  | V2 | V2 | 0.21 (0.03) |
| 6 |  | V2 | FFA | 0.19 (0.03) |  | V2 | VWFA | - |
| 7 |  | V2 | AMY | 0.40 (0.05) |  | V2 | AMY | - |
| 8 |  | FFA | V2 | -0.17 (0.04) |  | VWFA | V2 | - |
| 9 |  | FFA | FFA | 0.10 (0.04) |  | VWFA | VWFA | - |
| 10 |  | FFA | MFG | - |  | VWFA | IFG | - |
| 11 |  | FFA | AMY | - |  | VWFA | AMY | -0.15 (0.03) |
| 12 |  | MFG | FFA | -0.20 (0.03) |  | IFG | VWFA | -0.11 (0.03) |
| 13 |  | MFG | MFG | - |  | IFG | IFG | - |
| 14 |  | MFG | AMY | 0.13 (0.03) |  | IFG | AMY | 0.21 (0.04) |
| 15 |  | AMY | V1 | 1.24 (0.08) |  | AMY | V1 | - |
| 16 |  | AMY | V2 | 0.87 (0.06) |  | AMY | V2 | - |
| 17 |  | AMY | FFA | 1.45 (0.05) |  | AMY | VWFA | 0.49 (0.05) |
| 18 |  | AMY | MFG | 0.54 (0.03) |  | AMY | IFG | 0.24 (0.03) |
| 19 |  | AMY | AMY | -0.83 (0.04) |  | AMY | AMY | -0.58 (0.04) |

* Empty cells indicate parameters that do not have probability of being nonzero higher than 0.95. These parameters are considered not meaningful.

V1 = primary visual area; V2 = secondary visual area; IFG = inferior frontal gyrus; AMY = amygdala; VWFA = visual word form area; MFG = middle frontal gyrus; FFA = fusiform face area.

**2.1 Supplementary Figures**

**Supplementary Figure 1.** Details of the posterior parameter estimates for the **A** and **B** matrices for faces (left) and words (right). Only parameters with probability of being nonzero higher than 0.95 are displayed. Indexes of Figure A are indicated in Supplementary Table 2s. In Figure B, modulations of positive stimuli for V1, V2, FFA, MFG and AMY correspond to indexes 1, 2, 3, 4 and 5, respectively, while modulations of negative stimuli for V1, V2, FFA, MFG and AMY correspond to indexes 6, 7, 8, 9 and 10, respectively.

V1 = primary visual area; V2 = secondary visual area; IFG = inferior frontal gyrus; AMY = amygdala; VWFA = visual word form area; MFG = middle frontal gyrus; FFA = fusiform face area.
