# Supplementary figures and images for: Neural correlates of emotional valence for faces and words
Source: Front Psychol. 2023 Feb 23;14:1055054. doi: 10.3389/fpsyg.2023.1055054 (PMC9996044; doi:10.3389/fpsyg.2023.1055054)

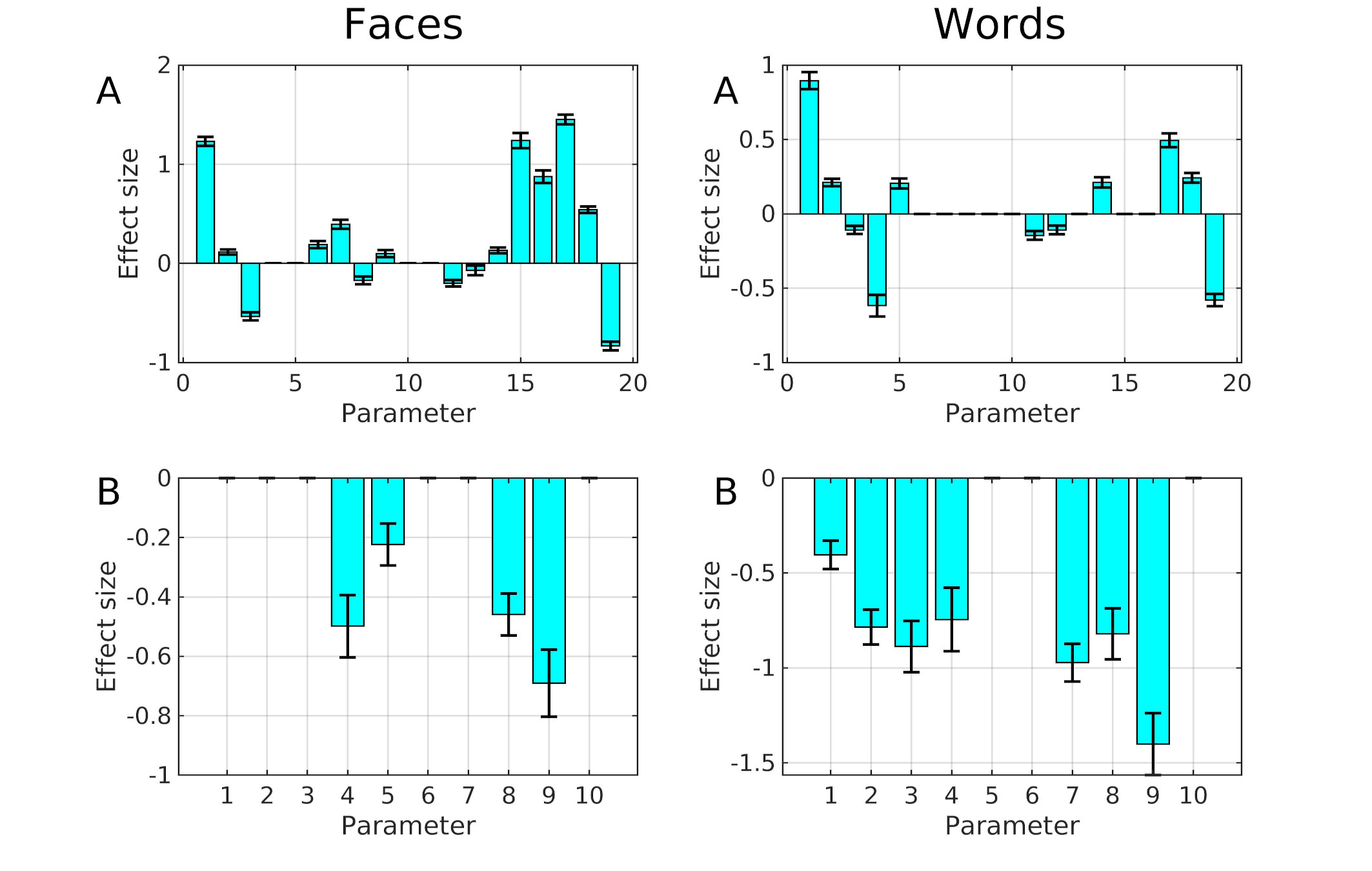

Supplement: Supplementary file 2 [file Image_1.JPEG]
